# Generation and analysis of spheroids from human primary skin myofibroblasts: an experimental system to study myofibroblasts deactivation

**DOI:** 10.1038/cddiscovery.2017.38

**Published:** 2017-07-17

**Authors:** Giuseppina Granato, Maria R Ruocco, Antonino Iaccarino, Stefania Masone, Gaetano Calì, Angelica Avagliano, Valentina Russo, Claudio Bellevicine, Gaetano Di Spigna, Giuseppe Fiume, Stefania Montagnani, Alessandro Arcucci

**Affiliations:** 1 Department of Public Health, University of Naples Federico II, Naples 80131, Italy; 2 Department of Molecular Medicine and Medical Biotechnology, University of Naples Federico II, Naples 80131, Italy; 3 Department of Clinical Medicine and Surgery, University of Naples Federico II, Naples 80131, Italy; 4 IEOS Istituto di Endocrinologia e Oncologia Sperimentale ‘G. Salvatore’, National Council of Research, Naples 80131, Italy; 5 Department of Translational Medical Sciences, University of Naples Federico II, Naples 80131, Italy; 6 Department of Experimental and Clinical Medicine, University of Catanzaro ‘Magna Graecia’, Viale Europa, Catanzaro 88100, Italy

## Abstract

Myofibroblasts are activated fibroblasts involved in tissue repair and cancer. They are characterized by *de novo* expression of *α*-smooth muscle actin (*α*-SMA), immunoregulatory phenotype and paracrine interaction with normal and tumorigenic cells leading to cell proliferation. At the end of wound-healing myofibroblasts undergo apoptotic cell death, whereas *in vitro*-activated fibroblasts are also subjected to a programmed necrosis-like cell death, termed nemosis, associated with cyclooxygenase-2 (COX-2) expression induction and inflammatory response. Furthermore, myofibroblasts form clusters during wound healing, fibrotic states and tumorigenesis. In this study, we generated and analysed clusters such as spheroids from human primary cutaneous myofibroblasts, which represent a part of stromal microenvironment better than established cell lines. Therefore, we evaluated apoptotic or necrotic cell death, inflammation and activation markers during myofibroblasts clustering. The spheroids formation did not trigger apoptosis, necrotic cell death and COX-2 protein induction. The significant decrease of *α*-SMA in protein extracts of spheroids, the cytostatic effect exerted by spheroids conditioned medium on both normal and cancer cell lines and the absence of proliferation marker Ki-67 after 72 h of three-dimensional culture indicated that myofibroblasts have undergone a deactivation process within spheroids. The cells of spheroids reverted to adhesion growth preserved their proliferation capability and can re-acquire a myofibroblastic phenotype. Moreover, the spontaneous formation of clusters on plastic and glass substrates suggests that aggregates formation could be a physiological feature of cutaneous myofibroblasts. This study represents an experimental model to analyse myofibroblasts deactivation and suggests that fibroblast clusters could be a cell reservoir regulating tissues turnover.

## Introduction

Human fibroblasts represent a very heterogeneous cell population, that in adult body exhibits embryonic and functional diversities.^1^ In fact, although most fibroblasts are considered mesodermal cells, some reports showed that fibroblasts of neck skin are derived from neural crest tissue.^2^ Fibroblasts maintain the homoeostasis of the extracellular matrix (ECM), but can also acquire an immunoregulatory phenotype.^2^ In fact, it is known that activated fibroblasts produce large amounts of cyclooxygenase-2 (COX-2) and proinflammatory cytokines, although the extent of fibroblast activation depends on the tissue type.^3^ Activated fibroblasts in healing wounds, fibrotic or cancer tissue express *de novo α*-smooth muscle actin (*α*-SMA), show increased levels of growth factors secretion and ECM-degrading proteases: these processes are regulated by inflammation and are involved in the differentiation of fibroblasts into myofibroblasts.^2,4–6^ At the end of wound healing, activated fibroblasts undergo apoptotic cell death, whereas *in vitro* fibroblasts endure also a programmed necrosis-like cell death, called nemosis.^7,8^ In particular, it is known that *in vitro* clusters of human dermal fibroblasts, named spheroids, are activated to produce massive amounts of COX-2, prostaglandins, proinflammatory cytokines and growth factors and thereby undergo nemosis associated with spheroids decomposition.^8^ It is noteworthy that a recent study showed that nemosis is a reversible process.^9^

Activated fibroblasts *in vivo* form clusters during wound-healing process, idiopathic pulmonary fibrosis and hypertrophic scars.^10,11^ Moreover, a recent work demonstrated that fibroblasts form aggregates in the dermis at early stages of melanoma development, before metastasis formation, and that a paracrine communication between cancer cells and fibroblasts leads to fibroblasts activation.^12^

Hence, the spheroids from human primary myofibroblasts could mimic regulation processes associated with myofibroblasts and tissues turnover.

The aim of this work was to produce and analyse spheroids from human primary myofibroblasts from normal skin of neck, evaluating apoptotic and necrotic cell death, inflammation and activation markers.

Our study demonstrated that spheroids formation is not associated with apoptotic or necrotic programmed cell death and that myofibroblasts, during clustering, have undergone a deactivation process. This work could represent a new experimental model to study myofibroblasts deactivation, and suggests an alternative process regulating the turnover of myofibroblasts and tissues.

## Results

### Analysis and clustering of human primary cells from neck skin

**Figure 1:**
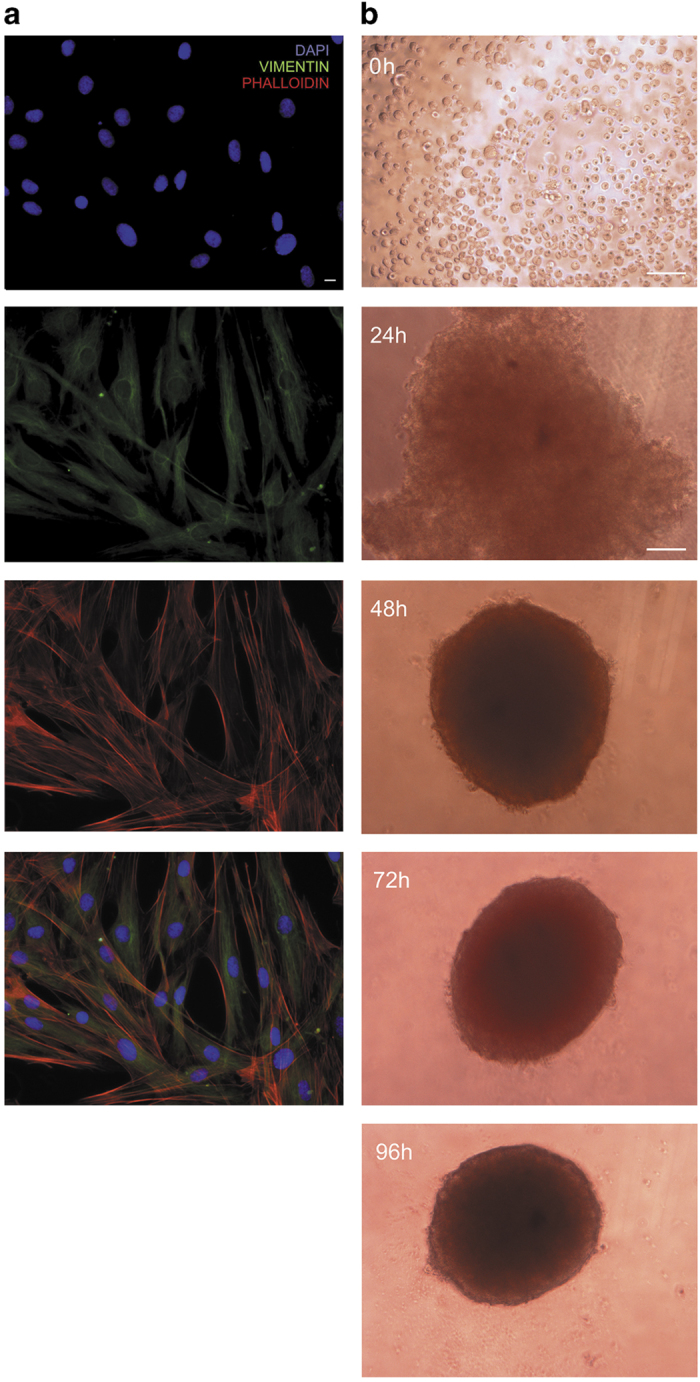
Analysis and clustering of human primary cells from neck skin. (**a**) Evaluation of vimentin immunofluorescence (green channel) and phalloidin staining (red channel) of primary cutaneous cells used to generate spheroids. DAPI (blue channel) was used to locate the nuclei of the cells (scale bar=10 *μ*m). Magnification ×40. (**b**) Photographs of cell-suspension drop at 0 h and of the spheroid during its three-dimensional culture. Photographs have been taken through phase contrast microscope at the indicated times (scale bar, 100 *μ*m). Magnification ×10. Volumes of spheroids during their maturation at 48, 72 and 96 h were 0.065 mm^3^±0.004, 0.053 mm^3^±0.003 and 0.043 mm^3^±0.003, respectively. Data are reported as means of three independent experiments±S.E. 96 h *versus* 48 h.

We first carried out the morphological analysis of cells obtained from normal neck skin and used to generate spheroids. To this aim, the presence of intermediate filaments and stress fibres was evaluated in monolayer cells by vimentin immunofluorescence and phalloidin staining analysis, respectively (Figure 1a). This investigation detected the presence of vimentin intermediate filaments and stress fibres that are cytoskeletal markers of proto-myofibroblasts, the cell type representing the intermediate step during fibroblasts to myofibroblasts differentiation process.^13^ Hence, we formed spheroids by using hanging-drops and agarose-coated U-bottom well plates adapted methods.^14^ We obtained multicellular aggregates 24 h after the seeding of fibroblasts drops into agarose-coated wells and spheroids only after 48 h (Figure 1b). The volume of spheroids during their maturation decreases after 96 h, as indicated in Figure 1b legend. This little decrease is due to a compaction process: in fact, spheroids did not undergo a decomposition process over time. We have cultured spheroids up to 216 h, as shown below.

**Figure 2:**
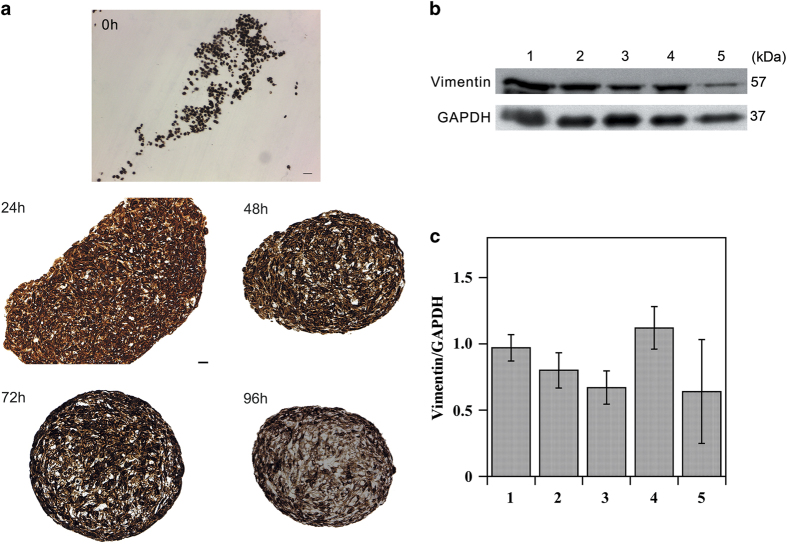
Analysis of vimentin. (**a**) Vimentin immunohistochemical analysis of paraffin-embedded sections of both cell-suspension drop at 0 h and spheroids collected at indicated time points (scale bar, 25 *μ*m). 0 h cell-suspension drop: Magnification ×10. Spheroids sections: Magnification ×20. All images are representative of three independent experiments. (**b**) Vimentin western blotting analysis of fibroblasts monolayer (1), spheroids collected at 24 h (2), 48 h (3), 72 h (4) and 96 h (5). GAPDH was used as loading control. Representative image of three independent experiments is shown. (**c**) Densitometric analysis of vimentin protein levels. Fibroblasts monolayer (1), spheroids collected at 24 h (2), 48 h (3), 72 h (4) and 96 h (5). Data are reported as means of three independent experiments±S.E.

Immunohistochemical analysis of paraffin-embedded sections of both cell-suspension drop at 0 h and spheroids collected at different times showed in cell-suspension drop and all spheroids the presence of vimentin (Figure 2a), an intermediate filament protein that is expressed in cells from mesenchymal origin.^15,16^ Western blotting analysis (Figures 2b and c) confirmed immunohistochemical results and did not show significant differences of vimentin protein levels in fibroblasts monolayer and spheroids.

### Terminal deoxynucleotidyl transferase-mediated nick end-labelling (TUNEL) assay for apoptosis detection

**Figure 3:**
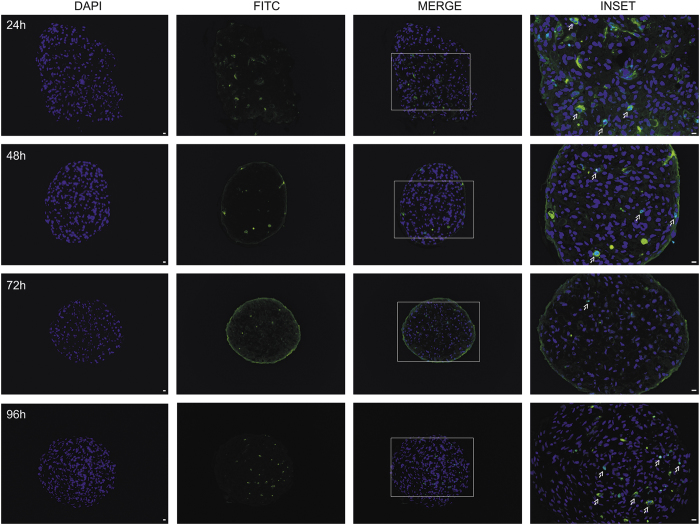
Identification of apoptotic cells. Terminal deoxynucleotidyl transferase-mediated nick end-labelling (TUNEL) assay for apoptosis detection in spheroids collected at different times (green channel). DAPI (blue channel) was used to locate the nuclei of fibroblasts (scale bar, 10 *μ*m). Magnification ×10. Inset: Magnification ×20. Apoptotic nuclei in the insets are indicated by arrows (scale bar, 10 *μ*m).

DNA fragmentation represents a characteristic hallmark of apoptosis and TUNEL can detect and assess apoptosis at single cell level, based on labelling of DNA strand breaks.^17^ Therefore, we performed TUNEL staining of sectioned spheroids to evaluate whether forcing adherent cells, such as fibroblasts, to grow as multicellular aggregates could trigger apoptotic cell death (Figure 3). This analysis evidenced a few nuclei positive for DNA strand breaks, which did not display a time-dependent increase. In particular, TUNEL staining of spheroids collected at different time points detected 2.3%, 1.6%, 0.47% and 0.22% of apoptotic cells at 24, 48, 72 and 96 h, respectively. These results indicated that fibroblasts clustering is not associated with apoptotic cell death.

### Evaluation of cell death markers

**Figure 4:**
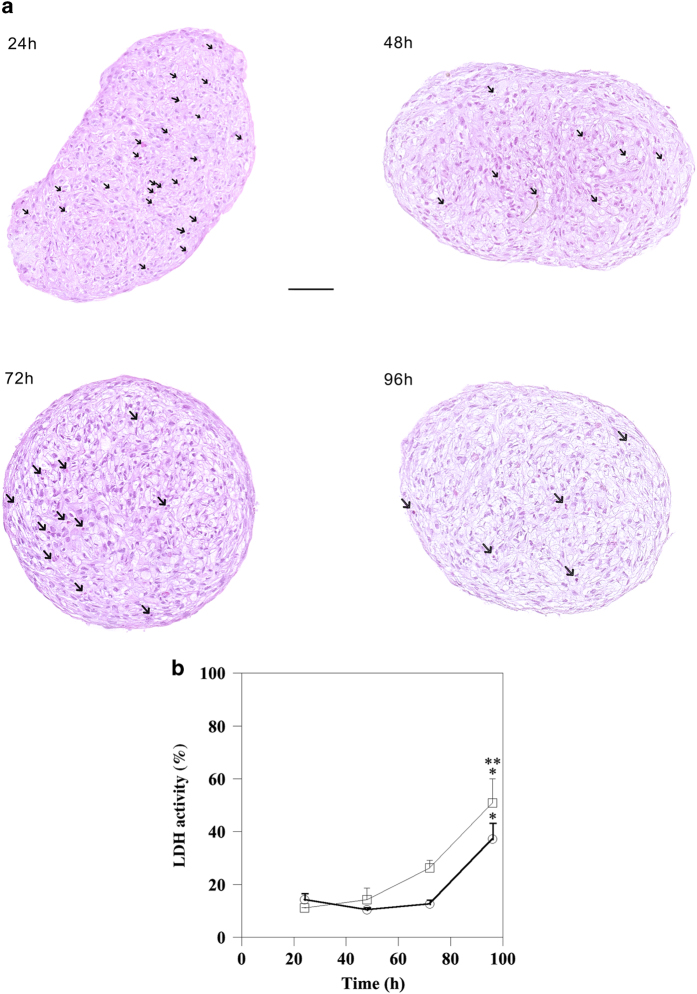
Cell death markers investigation. (**a**) Haematoxylin and eosin staining of paraffin-embedded sections of spheroids. The images of sections have been converted into high-resolution digital data (scale bar, 100 *μ*m). Magnification ×200. All images are representative of three independent experiments. The karyorrhectic nuclei, indicated by arrows, at 24 h, 48 h, 72 h and 96 h were: 22±2.1; 6.3±1.2; 6±2.5; 4±0.6, respectively. Data are reported as means of three independent experiments±S.E. *P*<0.005: 24 h *versus* 48 h and 72 h. *P*<0.001: 24 h *versus* 96 h. (**b**) At selected time points, necrosis was assessed by measurement of LDH activity released in the conditioned medium of both monolayers (squares) and spheroids (circles). Data are means of three independent experiments±S.E. In conditioned medium of monolayers: 96 h *versus* 24 h **P<0.005; 96 h *versus* 48 h *P<0.01. In conditioned medium of spheroids: 96 h *versus* 24 h, 48 h and 72 h *P<0.01.

Haematoxylin and eosin staining was performed to evaluate the presence of necrosis areas and apoptotic cells. This analysis did not detect necrosis in spheroids collected and analysed at different time points (Figure 4a). Moreover, spheroids stained with haematoxylin and eosin show some karyorrhectic nuclei, characteristic of apoptotic cell death and presenting a time-dependent decrease, indicated in Figure 4a legend. Therefore, haematoxylin and eosin staining of spheroids collected at different time points confirms the results of TUNEL assay. It is known that during necrosis loss of membrane integrity leads to cellular lactate dehydrogenase (LDH) release;^8^ hence, we measured, at indicated time points, LDH enzymatic activity in conditioned medium of fibroblasts monolayer or spheroids (Figure 4b). A comparable LDH activity was measured in conditioned medium of both monolayer and spheroid cultures. In particular, there is not a significant difference of LDH activity between conditioned medium of monolayer and spheroid collected at indicated time points. Conversely, a significant increase of LDH activity in the conditioned medium of monolayers or spheroids was detected only after 96 h, although more evident for myofibroblasts monolayer.

### Inflammation and activation markers levels

**Figure 5:**
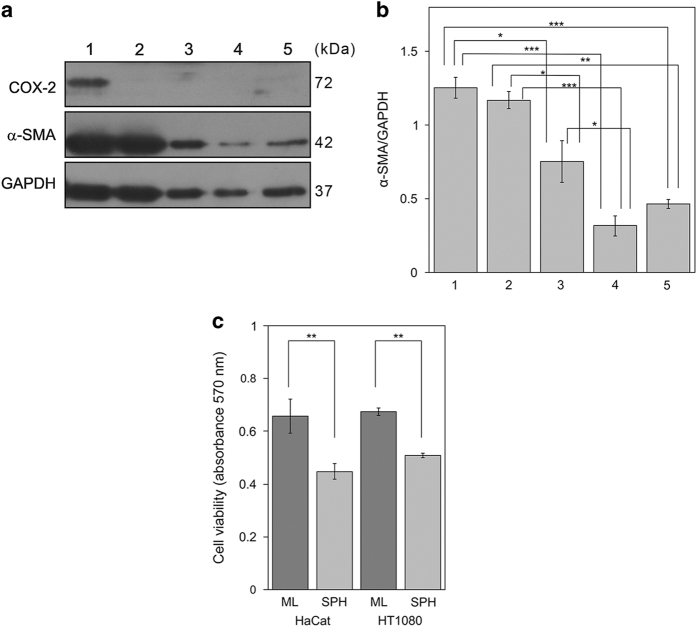
Inflammation and activation markers levels. (**a**) Western blotting analysis of COX-2 and *α*-SMA in protein extracts of fibroblasts monolayer (1), spheroids collected at 24 h (2), 48 h (3), 72 h (4) and 96 h (5). GAPDH was used as loading control. Representative image of three independent experiments is shown. (**b**) Densitometric analysis of *α*-SMA protein levels. Fibroblasts monolayer (1), spheroids collected at 24 h (2), 48 h (3), 72 h (4) and 96 h (5). Data are reported as means of three independent experiments ±S.E. *P<0.05, **P<0.005, ***P<0.0005. (**c**) Effect of conditioned medium of fibroblasts monolayer (ML) and spheroids (SPH) on normal (HaCat) and cancer (HT1080) cells. The cells were grown for 48 h with conditioned medium and cell viability was evaluated by MTT assay. Data are means of three independent experiments±S.E. **P<0.0001.

To evaluate the levels of inflammation and activation markers during fibroblasts clustering,^8,18^ we analysed, by western blotting, COX-2 and *α*-SMA proteins in extracts of fibroblasts monolayer and spheroids (Figure 5a). This analysis showed that the fibroblasts monolayer express COX-2 protein and that spheroids do not present COX-2 overexpression induction. Moreover, there is a reduction of *α*-SMA levels in spheroids protein extracts, compared with monolayer extracts. In particular, the densitometric analysis demonstrated a time-dependent *α*-SMA decrease already significant at 48 h and more evident at 72 and 96 h (Figure 5b). The expression of *α*-SMA (Figure 5a) together with the presence of vimentin intermediate filaments and stress fibres (Figure 1a) demonstrate that fibroblasts monolayer are myofibroblasts.^13^ The dramatic decrease of the *α*-SMA in spheroids collected at different times suggests that myofibroblasts underwent a deactivation process during clusters formation: in fact, the *α*-SMA is a specific marker of fibroblasts to myofibroblasts differentiation.^13,19^

It is known that activated fibroblasts enhance the proliferation of both normal and cancer cells, through paracrine interaction.^20^ Therefore, to further compare the activation state of myofibroblasts monolayer and spheroids, we analysed by MTT (3-(4,5-dimethylthiazole*-*2*-*yl)-2,5-biphenyltetrazolium bromide) assay the effects of monolayers and spheroids conditioned medium on viability and cell proliferation of non-malignant HaCat and fibrosarcoma HT1080 cell lines (Figure 5c).^20,21^ This analysis showed that the viability of both cell lines incubated with spheroids conditioned medium was clearly lower than the one of cells treated with monolayer conditioned medium. These data further indicate that myofibroblasts underwent a deactivation process in spheroids.

### Cell growth fraction in spheroids grown on agar or reverted to adhesion and monolayer growth

**Figure 6:**
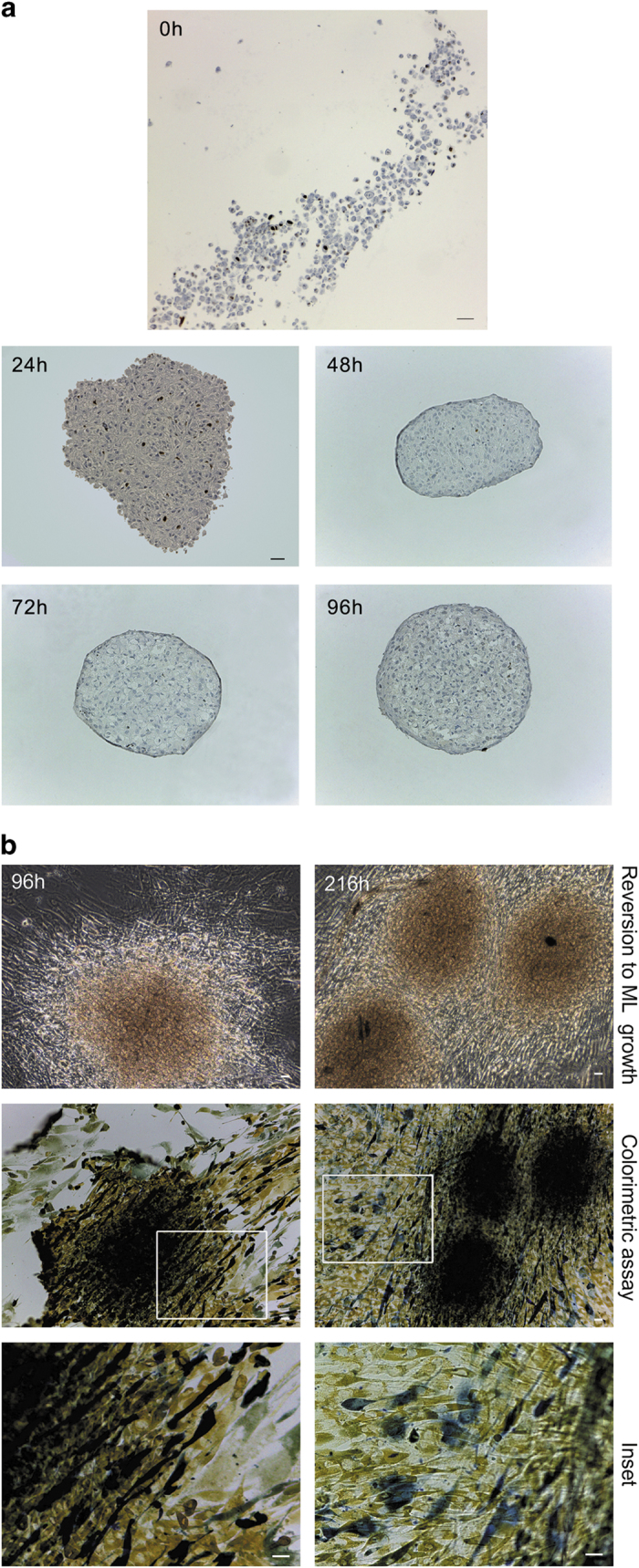
Evaluation of the cell growth fraction in spheroids grown on agar or reverted to adhesion and monolayer growth (ML). (**a**) Ki-67 immunohistochemical analysis of paraffin-embedded sections of myofibroblasts cell suspension at 0 h and spheroids collected at indicated time points (scale bar, 25 *μ*m). Myofibroblasts cell suspension at 0 h: Magnification ×10. Spheroids: magnification ×20. (**b**) Spheroids collected at 96 h and 216 h were transferred to plastic dishes and maintained in culture for 12 days before to be processed. Images represent spheroids reverted to adhesion growth, photographed by phase contrast microscope and then stained with a live cell-detection kit to monitor cell cycle. Yellow pixels represent cells in G0/G1 phase, green pixels represent cells in S phase and dark blue pixels represent cells in G2/M phase (scale bar, 25 *μ*m). Magnification ×10. Inset: Magnification ×20 (scale bar, 25 *μ*m). The images are representative of three independent experiments.

To evaluate the cell growth fraction in spheroids, we checked the presence of proliferation marker Ki-67 by immunohistochemical analysis (Figure 6a). This investigation revealed the presence of about 10% and 6% positive nuclei for Ki-67 only in myofibroblasts cell suspension at 0 h and spheroids collected at 24 h, respectively. In particular, Ki-67, in all spheroids sections tested, does not localize in a restricted area but is evident in the centre as well as in the external areas of spheroids. Moreover, at 48 h, we detected only one positive nucleus, while at 72 and 96 h, we did not detect any positive nucleus for Ki-67.

Spheroids grown on agar for 96 and 216 h were transferred on standard culture dishes to allow cell adhesion and spreading, and to analyse cells from spheroids during possible reversion to adhesion growth. This analysis demonstrated that spheroids can revert to adhesion growth (Figure 6b). Moreover, by a live cell-detection colorimetric assay, we evaluated cycle phases of cells outgrown from spheroids. This analysis showed that cells outgrowing from spheroids collected at 96 h were in G0/G1 (yellow pixels), S (green pixels) and G2/M (dark blue pixels) phases. Among all cell population derived from spheroids harvested at 216 h, there are still evident growing cells, although the ones in S phase are rare.

### Re-acquisition of myofibroblastic phenotype by cells of spheroids reverted to adhesion and monolayer growth

**Figure 7:**
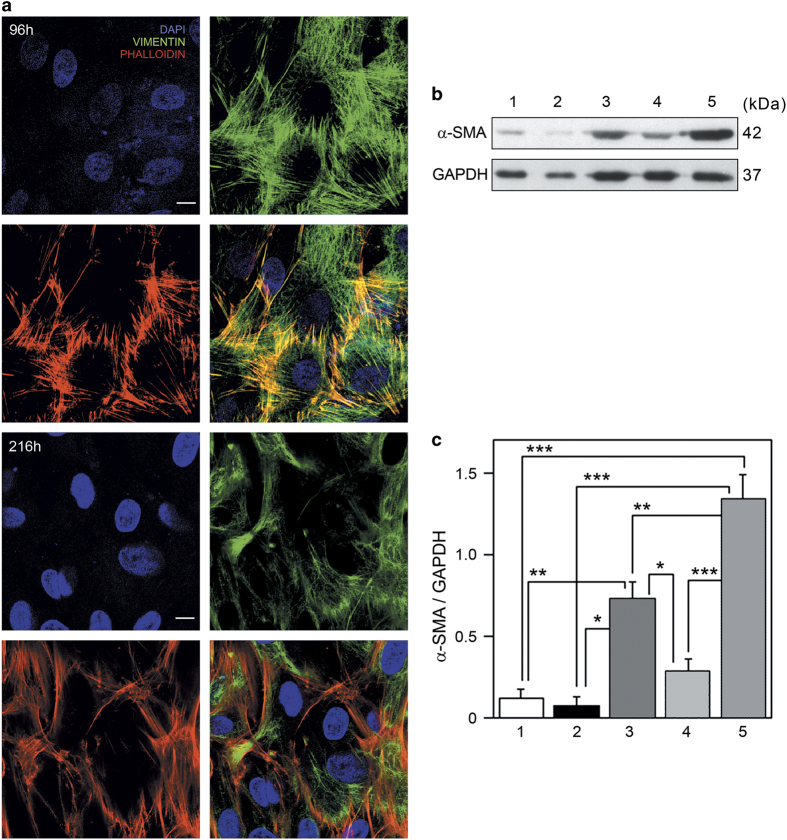
Spheroids collected at 96 h and 216 h were transferred to glass coverslips or plastic dishes, maintained in culture for 12 days and analysed. (**a**) Confocal fluorescence analysis of vimentin immunostaining (green channel) and phalloidin staining (red channel). DAPI (blue channel) was used to locate the nuclei (scale bar, 10 *μ*m). The images are representative of three independent experiments. (**b**) Western blotting analysis of *α*-SMA. Protein extracts of spheroids collected at 96 h (1), 216 h (2), spheroids reverted to adhesion growth after 96 h (3), 216 h (4) of three-dimensional culture and myofibroblasts monolayer (5). GAPDH was used as loading control. Representative image of three independent experiments is shown. (**c**) Densitometric analysis of *α*-SMA protein levels. Spheroids collected at 96 h (1), 216 h (2), spheroids reverted to adhesion growth after 96 h (3), 216 h (4) of three-dimensional culture and myofibroblasts monolayer (5). Data are reported as mean±S.E. *P<0.05, **P<0.01, ***P<0.0001.

Confocal immunofluorescence analysis of cells outgrowing from spheroids reverted to adhesion growth, showed that cells of spheroids collected at 96 h present vimentin intermediate filaments and stress fibres (Figure 7a). On the other hand, cells of spheroids collected at 216 h display evident vimentin intermediate filaments but present fewer stress fibres than spheroids collected at 96 h (Figure 7a). Furthermore, in spheroids collected at 96 h, a vimentin and phalloidin staining co-localization is evident (Figure 7a). Western blotting and densitometric analysis of *α*-SMA levels showed that there is a dramatic and significant decrease of the protein in spheroids collected at 216 h and reverted to adhesion growth, compared to myofibroblasts monolayer (Figures 7b and c). Conversely, spheroids collected at 96 h and reverted to adhesion growth display *α*-SMA increased proteins level compared either with spheroids grown on agar and spheroids reverted to adhesion growth after 216 h of three-dimensional culture. Therefore, immunofluorescence and western blotting analysis indicate that deactivation process could be reversible for spheroids collected at 96 h because they present stress fibres and express *α*-SMA again. The spheroids collected at 216 h, instead, present stress fibres less developed and display low *α*-SMA levels, comparable to spheroids grown only on agar.

### Clusters and spheroids formed spontaneously on plastic or glass substrate

**Figure 8:**
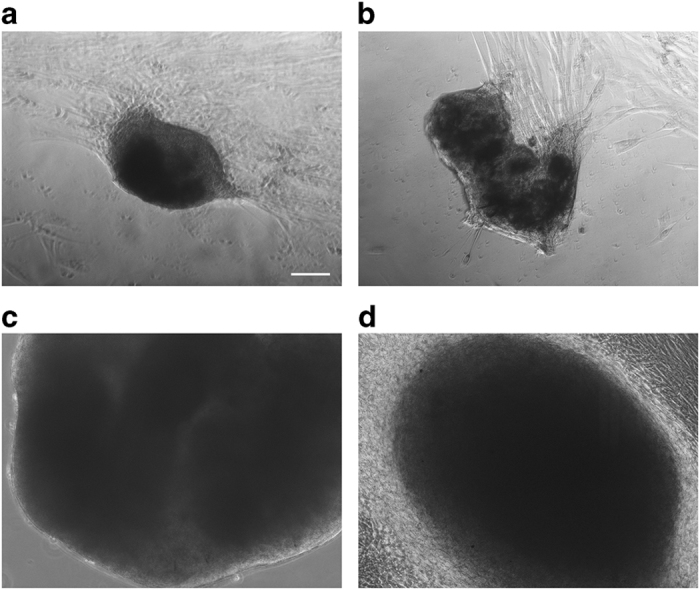
Clusters and spheroids formed spontaneously, without using hanging-drops and agarose-coated U-bottom well plates methods. (**a** and **b**) Clusters formed on the bottom of plastic 12-well plates. (**c** and **d**) Spheroid formed on glass coverslip and reverted to adhesion growth (scale bar, 100 *μ*m). Magnification ×10.

Surprisingly, we found that cutaneous myofibroblasts can form multicellular aggregates spontaneously, in absence of agar, both on plastic 12-well plates and on glass coverslips, 16 days after the seeding of cells (Figure 8). In particular, fibroblasts originated clusters and spheroids when the cell cultures were 100% confluent for several days. Furthermore, the outgrowth of cells from spheroid, reverted to adhesion growth, clearly demonstrates the vitality of cells inside spheroids (Figure 8d). Hence, these data suggest that the formation of clusters could be a physiological feature of these cells.

## Discussion

The fibroblast population of adult body shows functional differences associated with diversities in embryonic origins.^1,2^ In particular, some authors consider neck skin fibroblasts originated from neural crest tissue, although most fibroblasts are considered mesodermal cells.^2^

In our experimental system, cells used to form spheroids are myofibroblasts from normal neck skin. Fibroblasts *in vitro* differentiate to myofibroblasts when cultured on high stiffness substrate, such as plastic dishes, in the presence of TGF-*β*.^13^ Furthermore, Santiago *et al*.^22^ showed that cardiac fibroblasts, cultured on rigid matrix represented by standard plastic plates, express *α*-SMA and differentiate to myofibroblasts. The myofibroblats used to generate spheroids express COX-2 protein, and this result is in agreement with a previous study showing that fetal bovine serum, present in fibroblasts monolayer and spheroids culture medium, can induce COX-2 expression and activate a transcriptional programme related to physiology of wound repair.^23^

This study demonstrated that human primary myofibroblasts from neck skin, when forced to grow as multicellular aggregates, do not undergo apoptosis or nemosis. Furthermore, vimentin immunohistochemical and western blotting analysis demonstrated that during spheroids formation fibroblasts save their mesenchymal origin. We showed that cell clustering does not lead to induction of COX-2 protein and spheroids decomposition, typical features of nemosis.^8^ COX-2 protein mediates tissue inflammation and tumorigenesis.^24^ In particular, as a result of tissue injury, fibroblasts migrate to the injured sites where they change to an activated and proinflammatory phenotype, associated with COX-2 expression.^24^ In our experimental system, the lack of COX-2 protein overexpression, during spheroids formation, suggests that myofibroblasts aggregation is not associated with inflammatory response.

Moreover, the significant decrease of *α*-SMA protein levels, the most widely used molecular marker of myofibroblasts,^19,25^ indicates that three-dimensional aggregation of myofibroblasts induced a deactivation process.

Analysis of reverted spheroids showed that this deactivation process is reversible for spheroids collected at 96 h and seems to be partially reversible for spheroids collected at 216 h. In fact, spheroids collected at 216 h developed stress fibres less extensively than spheroids collected at 96 h. Moreover, the significant difference of *α*-SMA levels between spheroids reverted to adhesion growth after 96 and 216 h of three-dimensional culture, confirms and explains the immunofluorescence analysis. Therefore, the morphological and molecular phenotype of spheroids collected at 216 h resembles proto-myofibroblasts phenotype.^13^

Vimentin and phalloidin staining co-localization detected only in spheroids collected at 96 h is explained by their higher phalloidin staining, compared with spheroids collected at 216 h, and it is in agreement with previous studies showing that stress fibres contain vimentin and that actin and vimentin filaments can interact directly.^26,27^

The deactivation of myofibroblasts during clusters formation is confirmed by MTT assay. Previous study demonstrated that conditioned medium of activated fibroblasts within spheroids triggers the proliferation of nontumorigenic and tumorigenic cell lines.^20^ Conversely, in our experimental system, the cytostatic effect exerted by conditioned medium of spheroids on normal HaCat and metastatic HT1080 cell lines is evident. Paracrine epithelial–mesenchymal cell interactions have an important role in both tissue repair and cancer progression, through increased secretion of growth factors that support the proliferation of normal and tumorigenic cells.^3,4,19,20,28^ Our results, instead, indicate that the spheroids from primary cutaneous myofibroblasts do not resemble stromal microenvironment typical of wound healing and cancer.

Moreover, the deactivation of myofibroblasts inside spheroids is also supported by Ki-67 immunohistochemical analysis showing that in spheroids collected at 72 and 96 h there are no positive nuclei for Ki-67 and all the cells are in G_0_ cycle phase: in fact, it is known that Ki-67 expression is associated with cell cycle progression.^29^ The immunohistochemical evaluation of Ki-67 and the *α*-SMA western blotting analysis of spheroids suggests that myofibroblasts deactivation is a step by step process.

However, colorimetric evaluation of cycle phases of cells from spheroids reverted to adhesion growth demonstrated that fibroblasts keep their proliferation capability within spheroids.

For many years, it was supposed that myofibroblasts could not revert to inactivated fibroblasts and myofibroblasts were considered cells terminally differentiated. In particular, at the end of wound healing, myofibroblasts undergo programmed cell death and to date it is not clear whether they can acquire a stable inactivated phenotype *in vivo*.^7,30,31^ It is becoming evident that myofibroblasts can revert to a non-activated phenotype.^32^ In fact, it has been shown that murine myofibroblasts, originated from hepatic stellate cells, could revert to inactivated phenotype during regression of liver fibrosis: these deactivated cells can be rapidly reactivated into myofibroblasts in response to fibrogenic stimuli.^33^

Our report shows that forcing myofibroblasts to grown as multicellular aggregates induces myofibroblasts deactivation, and suggests an alternative process regulating the turnover of myofibroblasts. It is noteworthy that we have observed that primary cutaneous myofibroblasts are able to form spheroids spontaneously on high stiffness substrates represented by plastic or glass. It will be interesting to analyse these spontaneous spheroids and evaluate differences and resemblances with spheroids generated on agar.

The growth of fibroblasts as spheroids can be associated with conditions where fibroblasts lose their contact to connective tissue. In particular, fibroblasts can lose their contacts to ECM during inflammation, wound healing and cancer. In fact, in these situations several proteases, destroying ECM structure, liberate fibroblasts from ECM and trigger fibroblasts clusters formation.^9^

Moreover, another work detected fibroblasts aggregates in the dermis at early stages of melanoma development, before metastasis formation and showed a paracrine communication between cancer cells and fibroblasts.^12^

In conclusion, this study demonstrated that spheroids from human cutaneous primary myofibroblasts can be maintained in culture and that clusters formation does not trigger programmed cell death, but could represent a reservoir of fibroblasts. Our work highlights new aspects about myofibroblasts turnover and could contribute to better understating the interaction between stroma and both normal and cancer cells.

## Materials and methods

### Cutaneous tissues

Normal skin specimens were obtained from donors (*n*=7, females, mean age 50±2.5) who had undergone neck surgery for benign pathologies. Patients with metabolic and connective tissue diseases were excluded. The investigation conforms to the principles outlined in the Declaration of Helsinki^34^ and informed consent was obtained from all the patients. The study reported in the manuscript has received the approval from the Ethic Committee of the University of Naples Federico II (Comitato Etico Università Federico II). The assigned protocol number of the study is 172/16.

### Cell cultures

Cutaneous fibroblasts were obtained as follows: surgical fragments were cut and after repeated PBS washing, were plated and cultured in Dulbecco’s minimal essential medium (DMEM; Sigma-Aldrich, Saint Louis, MO, USA) containing 200 mM l-glutamine, penicillin (100 mg/ml), streptomycin (100 mg/ml) and 10% FBS (GIBCO, Grand Island, NY, USA). The plates were incubated at 37 °C in the presence of 5% CO_2_ and the medium was removed every 3 days. The outgrowth of fibroblasts from surgical fragments was observed after 1 week. When fibroblasts were 75% confluent, surgical fragments were removed and the cells were detached with 0.25% trypsin-EDTA and replated. All the experiments were performed only with cells from early passage (<8).

Human keratinocyte (HaCat) and human metastatic fibrosarcoma cell lines (HT1080) were kindly provided by CEINGE (Naples, Italy).

The cells and spheroids were observed with a phase contrast microscope Olympus (Tokyo, Japan, Asia) CKX41 model; the images were acquired with a camera connected to the microscope by means Cell-A software.

All other chemicals were of analytical grade and were purchased from Sigma-Aldrich.

### Immunofluorescence analysis

Morphological analysis of fibroblasts was performed by plating the cells on glass coverslips, fixing with 4% paraformaldehyde, permeabilizing with 0.1% Triton X-100 and blocking in donkey serum (Millipore, Billerica, MA, USA), diluted 1 : 10 in 1× PBS, for 30 min at room temperature. Glass coverslips were incubated with a mouse monoclonal anti-vimentin primary antibody (Sigma-Aldrich), diluted 1 : 50, for 1 h at 37 °C, and then washed three times with 1× PBS and subsequently incubated with a FITC donkey anti-mouse secondary antibody (Jackson ImmunoResearch, Suffolk, UK) diluted 1 : 50 and phalloidin-TRITC (Sigma-Aldrich) diluted 1 : 100, for 1 h at 37 °C. The cell nuclei were labelled with DAPI (Vector Laboratories, Inc, Burlingame, CA, USA). Glass coverslips mounting was done in Vectashield (Vector Laboratories). The images were taken with digital camera connected to the microscope (Leica DFC345FX, Leica Microsystems, Wetzlar, Germany) and then merged with the software Leica Application Suite 3.6.

Spheroids reverted to monolayer growth, previously fixed with 4% paraformaldehyde, were washed twice in 50 mM NH_4_Cl, permeabilized for 5 min with 0.2% Triton X-100 as reported by Mascia *et al.*^35^

The nuclei were stained with DAPI. Immunofluorescence analysis was performed with a confocal laser scanning microscope LSM 700 (Zeiss, Gottingen, Germany) equipped with three lasers whose wavelengths are 405, 488 and 555 nm. Fluorescence emission was revealed by 460–489 band pass filter for DAPI; 505**–**530 band pass filter for FITC and by 560**–**615 band pass filter for TRITC. Triple-staining immunofluorescence images were acquired separately in the green, red and UV channels at a resolution of 102×1024 pixels, with the confocal pinhole set to one Airy unit and then saved in TIFF format.

All other chemicals were of analytical grade and were purchased from Sigma-Aldrich.

### Generation and growth of spheroids

To obtain spheroids, we used the previously described hanging-drops and agarose-coated U-bottom well plates adapted methods.^14^ To this aim, fibroblasts were detached from culture dishes by trypsin and 20 *μ*l of cell-suspension drop, containing 1×10^4^ cells, were put on the lids of 96-well plates, containing 100 *μ*l of 1× PBS to avoid culture medium evaporation. After 12 h of incubation at 37 °C, the drops were transferred to U-bottom 96-well plates, containing 80 *μ*l of complete DMEM, and previously coated with 1% agarose (Applichem, Gatersleben, Saxony-Anhalt, Germany) in 1× PBS to avoid cell plate adhesion. Spontaneous spheroids were originated by plating 5×10^4^ cells onto bottom of 12-well plates or on glass coverslips and maintained in culture for several days. In particular, after 16 days, in many wells of plates, clusters and spheroids were evident.

The volumes of spheroids were calculated as previously described.^14^

The detection of cycle phases of cells from spheroids reverted to adhesion growth was performed, according to the manufacturer’s protocol, through Cell-Clock Mammalian Cell Cycle Assay (Biocolor, Antrim, UK).

### Immunohistochemistry

Each spheroid was fixed in 10% neutral buffered formalin and embedded in paraffin (Bio-Optica Milano SpA, Milan, Italy), then sliced into serial 4 *μ*m-thick sections and placed on poly-l-lysine coated glass slides (Menzel-Glaser, Brunswick, Germany). The slides were deparaffinized, rehydrated and immersed in 10 mM citric acid pH 6 (Sigma-Aldrich), in a microwave oven (VWR International PBI Srl, Milan, Italy) for three cycles of 5 min at 650 Watt, to exclude epitope masking owing to fixation. Spheroids sections were haematoxylin and eosin stained for necrosis and apoptosis analysis. Therefore, these sections, on glass slides, were converted into high-resolution digital data through NanoZoomer-2.0RS digital scanner (Hamamatsu, Tokyo, Japan, Asia). The preparation of paraffin-embedded cell suspensions were performed by using Shandon Cytoblock Cell Block Preparation System (Thermo Scientific, Rockford, IL, USA) according to the manufacturer’s protocol.

Other sections were immunostained with primary antibodies against Vimentin or Ki-67 (Ventana Medical Systems, Tucson, AZ, USA) detected by ULTRA View UNIVERSAL DAB DETECTION KIT (Ventana Medical Systems), according to the manufacturer’s protocol. Microscopy analysis was performed with a Leica DMLB microscope (Leica Microsystems). The images were taken in bright field with a digital camera (Leica DC200; Leica Microsystems) connected to the microscope.

### TUNEL and LDH assays

TUNEL staining was performed on spheroids sections, previously deparaffinized twice in xylene and rehydrated in graded series of ethanol, using ApopTag Plus Fluorescein In Situ Apoptosis Detection Kit (Millipore), according to the manufacturer’s protocol.

To perform LDH enzymatic assay, 1×10^4^ cutaneous myofibroblasts/well were grown as monolayer or spheroid cultures and, at selected time points, cytotoxicity was evaluated by measurement of LDH activity in the culture medium. Briefly, various aliquots of cell incubation medium were added to 1 ml reaction mixture containing 0.1 M Tris-HCl, pH 7.5, 125 *μ*M NADH. The reaction started with the addition of 600 *μ*M sodium pyruvate followed by the decrease in absorbance at 340 nm. The results were normalized respect to 100% death caused by various freezing and thawing of fibroblasts.

All other chemicals were of analytical grade and were purchased from Sigma-Aldrich.

### Total cell lysates and western blotting analysis

Monolayer cells and spheroids, at indicated time points, were collected, washed with PBS, lysed in ice-cold modified RIPA buffer containing 50 mM Tris-HCl, pH 7.4, 150 mM NaCl, 1% Nonidet P-40, 0.25% sodium deoxycholate, 1 mM Na_3_VO_4_ and 1 mM NaF supplemented with Protease Inhibitor Cocktails (Roche Diagnostics Corporation, Mannheim, Germany) and incubated for 30 min on ice. The supernatant, obtained after centrifugation at 12 000 *g* for 20 min at 4 °C, constituted total protein extract. Protein concentration was determined by Bradford’s method, using bovine serum albumin as calibrator.^36^ Western blotting analysis was performed with equal amounts of total protein extracts (20 *μ*g). Briefly, protein samples were dissolved in SDS-reducing loading buffer, run on a 10% SDS/PAGE at 120 V and then transferred to Immobilon P membrane (Millipore). The membranes were incubated overnight at 4 °C with the following primary antibodies: COX-2, *α*-SMA (Abcam, Cambridge, UK), Vimentin (Cell Signal Technology Inc., Danvers, MA, USA) and GAPDH (Cell Signal Technology) and then with horseradish peroxidase-linked specific secondary antibodies (Santa Cruz Biotechnology, Santa Cruz, CA, USA) at room temperature for 1 h. The membranes were analysed by an enhanced chemiluminescence reaction, using Super Signal West Femto Maximum Sensitivity Substrate kit (Thermo Scientific) according to the manufacturer's instructions. The signals were visualized by autoradiography. The images were acquired by Epson Perfection 2480 Photo (Epson, Suwa, Japan) and densitometric analysis was performed using the Image J 1.48i version (Wayne Rasband National Institutes of Health, USA).

All other chemicals were of analytical grade and were purchased from Sigma-Aldrich.

### MTT assay

To evaluate the effect of monolayers or spheroids conditioned medium on normal HaCaT and tumorigenic HT1080 cell lines, 1×10^4^ cells/well were seeded on 96-well plates and let to grow for 24 h. Then, the culture medium was removed and replaced with 100 *μ*l of conditioned medium from monolayers or spheroids, cultured for 72 h. After 48 h of incubation, MTT cell viability assay was performed. Briefly, 10 *μ*l of 12 mM MTT solution was added to each well, incubated for 3 h at 37 °C in the dark and then MTT formazan crystals were dissolved in 100 *μ*l of solubilization solution (0.01 M HCl in isopropanol). Cells number is correlated with the amount of MTT formazan formed, that was evaluated by measuring absorbance at 570 nm, using Multiscan EX microplate reader (Thermo Labsystems, Vantaa, Finland).

All chemicals were of analytical grade and were purchased from Sigma-Aldrich.

### Statistical analysis

Numerical data were reported in Kaleida Grafh 4.0 and analysed by Student’s *t*-test; one-way ANOVA, with Bonferroni corrections, was used for multiple comparisons.
